# Supplementary material for: Editing stem cell genomes at scale to measure variant effects in diverse cell and genetic contexts
Source: medRxiv. 2025 Nov 14:2025.11.12.25340127. Preprint. [Version 1] doi: 10.1101/2025.11.12.25340127 (PMC12642741; doi:10.1101/2025.11.12.25340127)

medRxiv preprint doi: https://doi.org/10.1101/2025.11.12.25340127; this version posted November 14, 2025. The copyright holder for this preprint (which was not certified by peer review) is the author/funder, who has granted medRxiv a license to display the preprint in perpetuity.

It is made available under a CC-BY 4.0 International license .

Supplementary Figure 1:

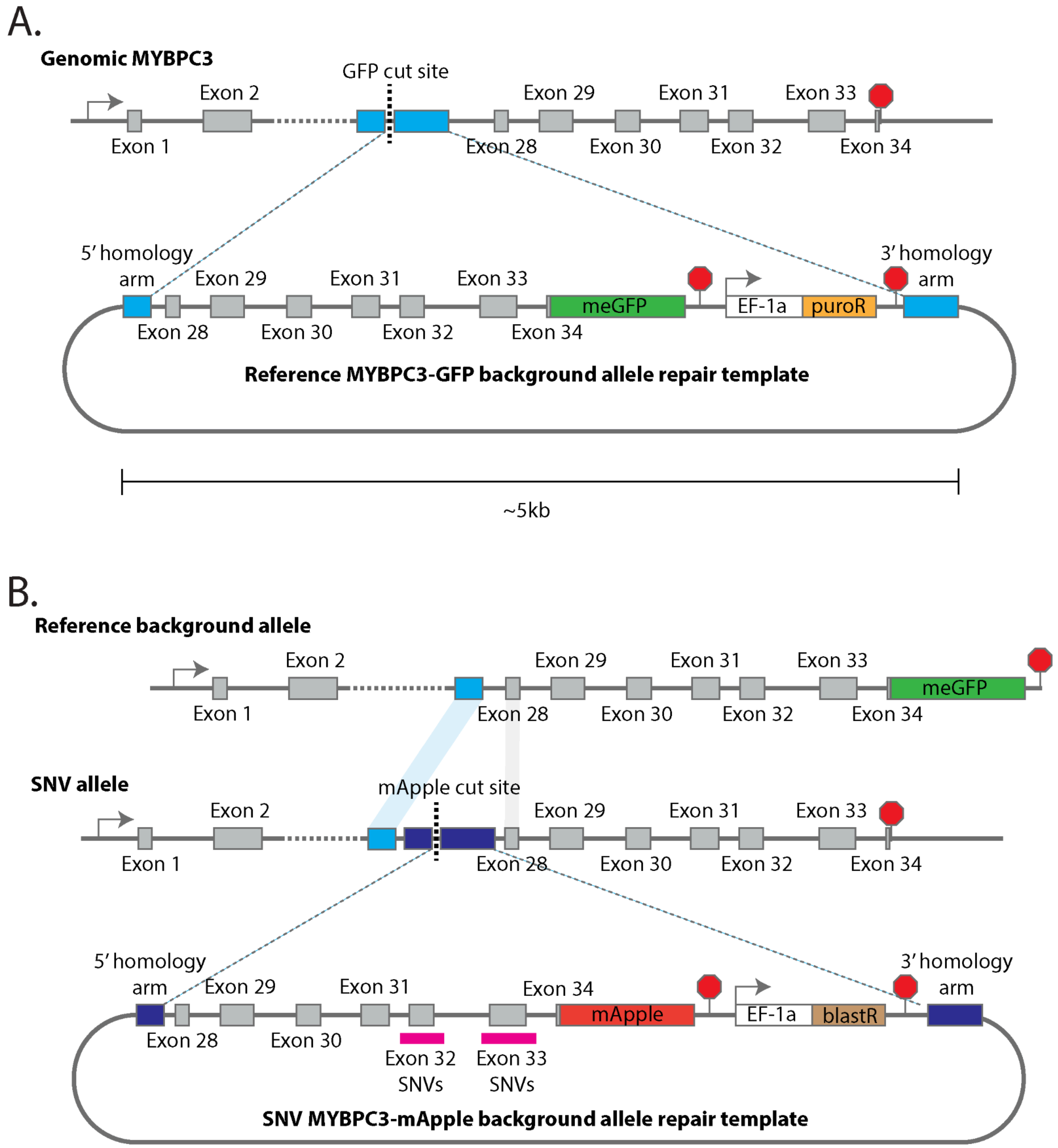

medRxiv preprint doi: https://doi.org/10.1101/2025.11.12.25340127; this version posted November 14, 2025. The copyright holder for this preprint (which was not certified by peer review) is the author/funder, who has granted medRxiv a license to display the preprint in perpetuity.

It is made available under a CC-BY 4.0 International license .

Supplementary Figure 2:

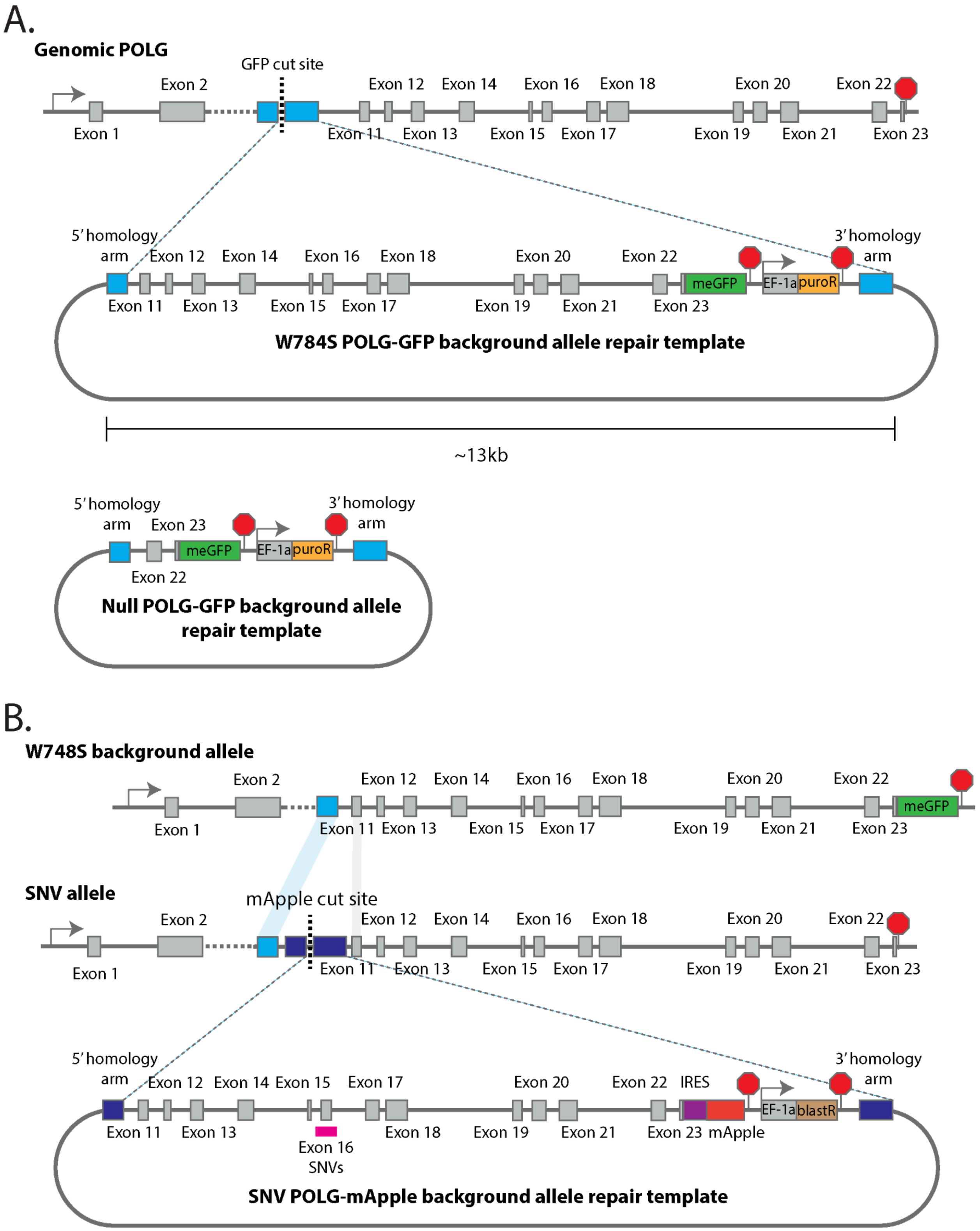

medRxiv preprint doi: https://doi.org/10.1101/2025.11.12.25340127; this version posted November 14, 2025. The copyright holder for this preprint (which was not certified by peer review) is the author/funder, who has granted medRxiv a license to display the preprint in perpetuity.

It is made available under a CC-BY 4.0 International license .

Supplementary Figure 3:

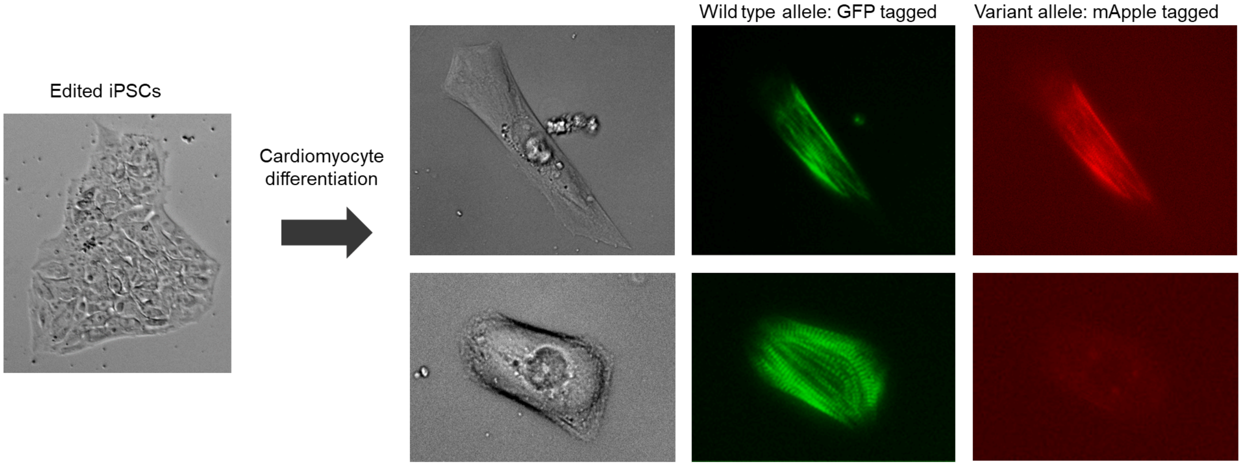

Supplementary Figure 4:

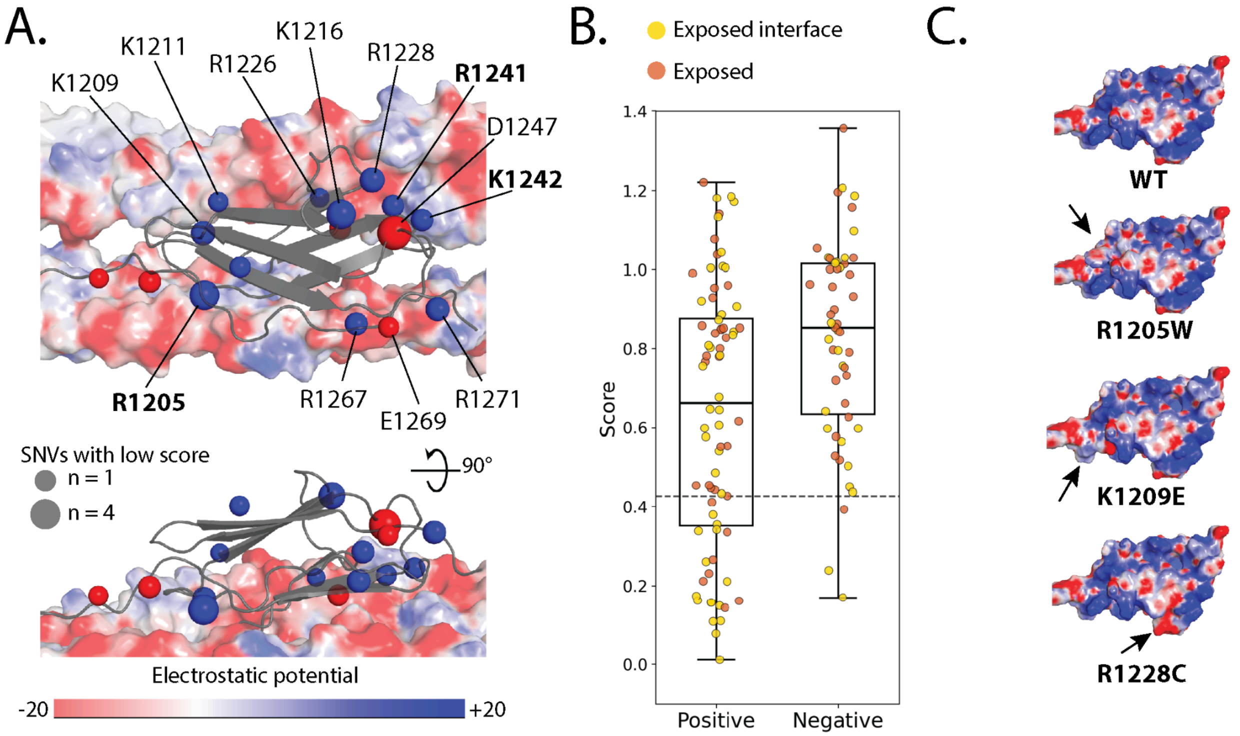

medRxiv preprint doi: https://doi.org/10.1101/2025.11.12.25340127; this version posted November 14, 2025. The copyright holder for this preprint (which was not certified by peer review) is the author/funder, who has granted medRxiv a license to display the preprint in perpetuity.

It is made available under a CC-BY 4.0 International license .

Supplementary Figure 5:

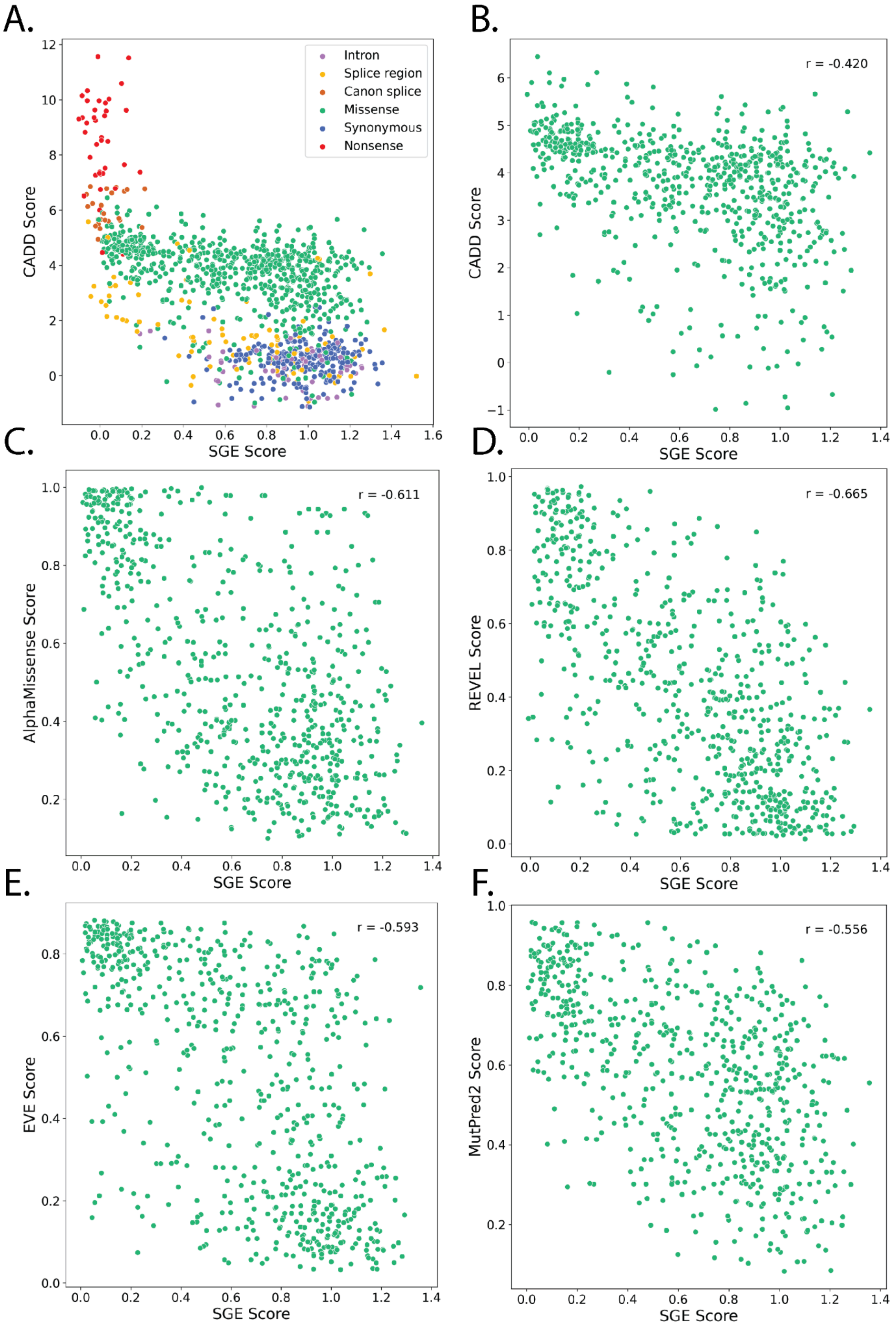

medRxiv preprint doi: https://doi.org/10.1101/2025.11.12.25340127; this version posted November 14, 2025. The copyright holder for this preprint (which was not certified by peer review) is the author/funder, who has granted medRxiv a license to display the preprint in perpetuity.

It is made available under a CC-BY 4.0 International license .

Supplementary Figure 6:

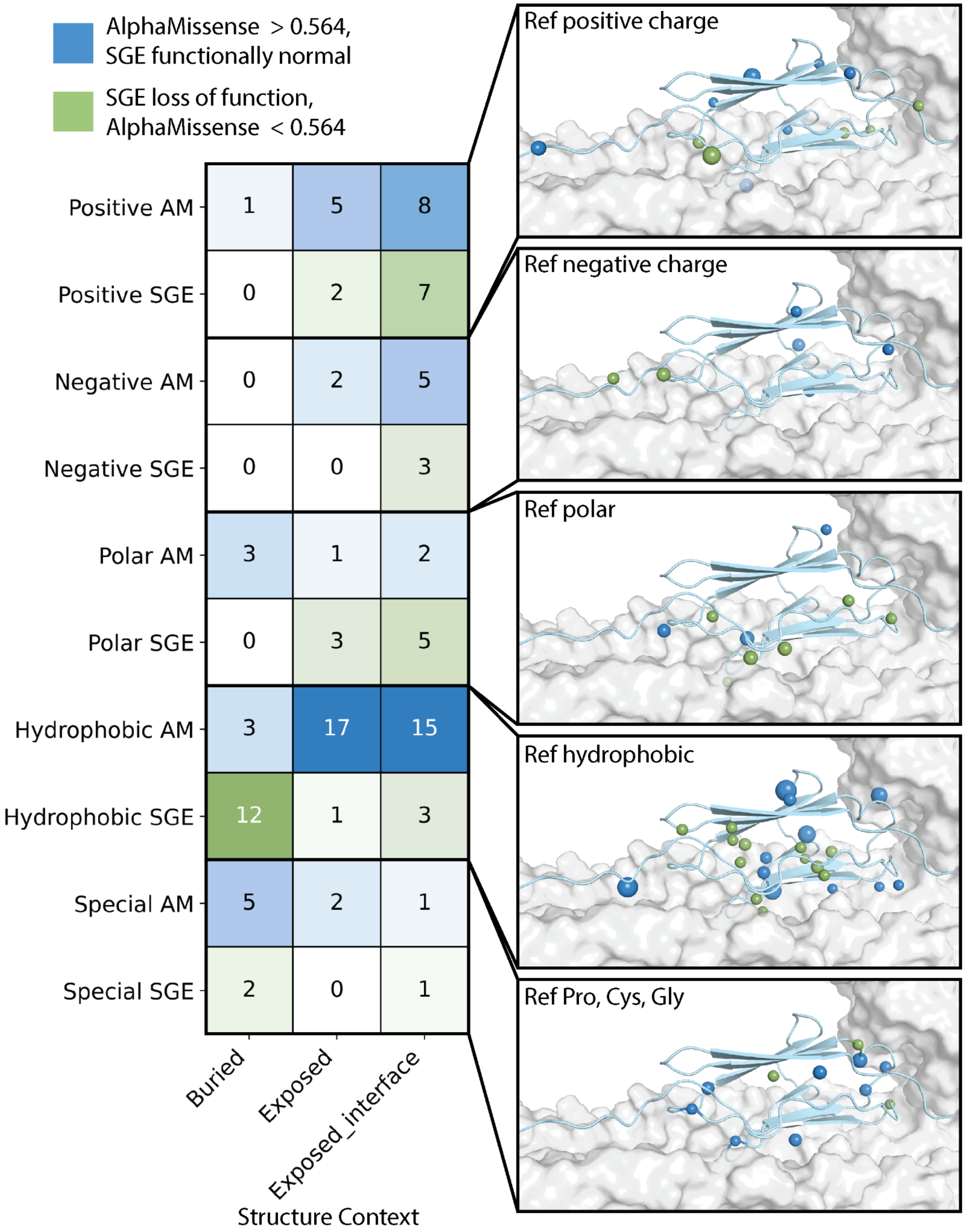

medRxiv preprint doi: https://doi.org/10.1101/2025.11.12.25340127; this version posted November 14, 2025. The copyright holder for this preprint (which was not certified by peer review) is the author/funder, who has granted medRxiv a license to display the preprint in perpetuity.

It is made available under a CC-BY 4.0 International license .

Supplementary Figure 7:

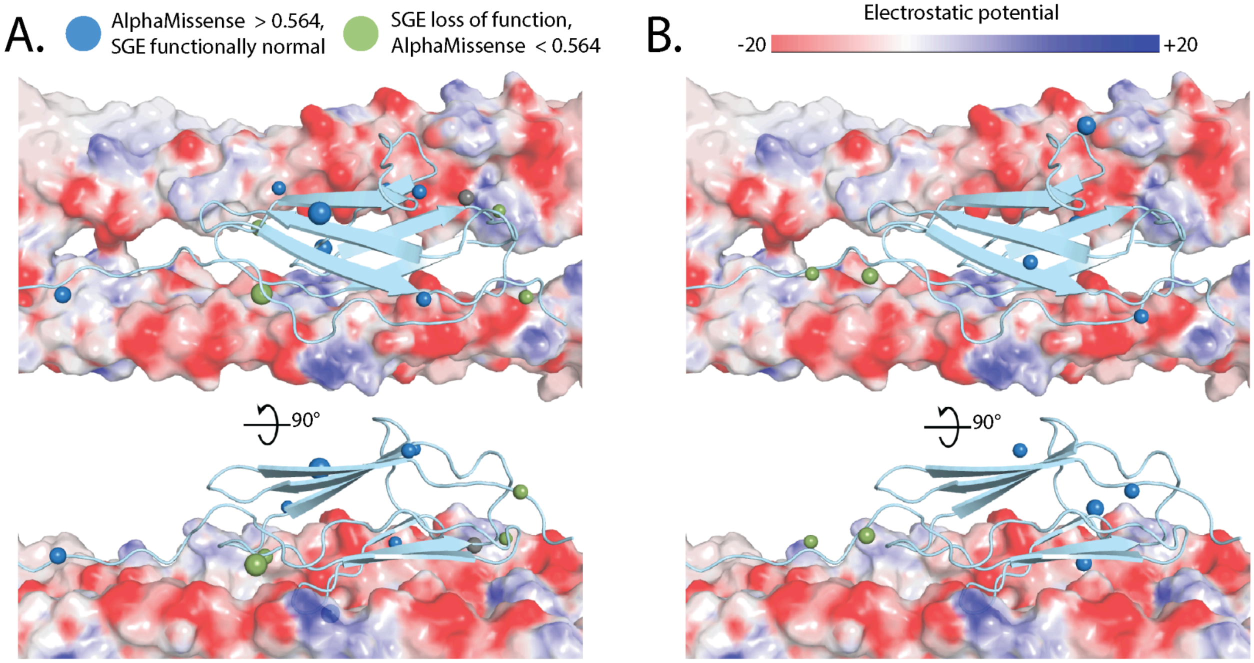

medRxiv preprint doi: https://doi.org/10.1101/2025.11.12.25340127; this version posted November 14, 2025. The copyright holder for this preprint (which was not certified by peer review) is the author/funder, who has granted medRxiv a license to display the preprint in perpetuity.

It is made available under a CC-BY 4.0 International license .

Supplementary Figure 8:

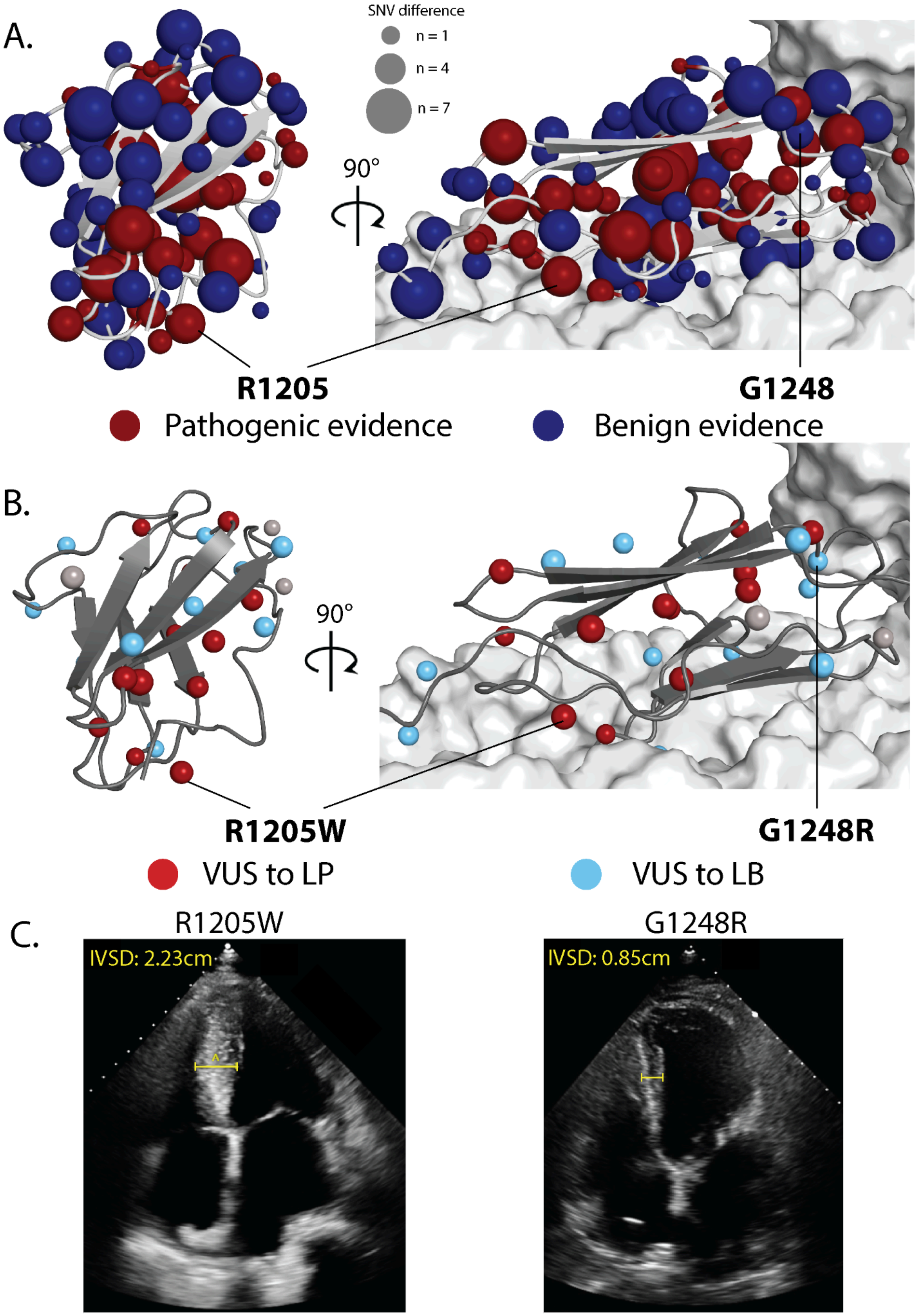

medRxiv preprint doi: https://doi.org/10.1101/2025.11.12.25340127; this version posted November 14, 2025. The copyright holder for this preprint (which was not certified by peer review) is the author/funder, who has granted medRxiv a license to display the preprint in perpetuity.

It is made available under a CC-BY 4.0 International license .

Supplementary Figure 9:

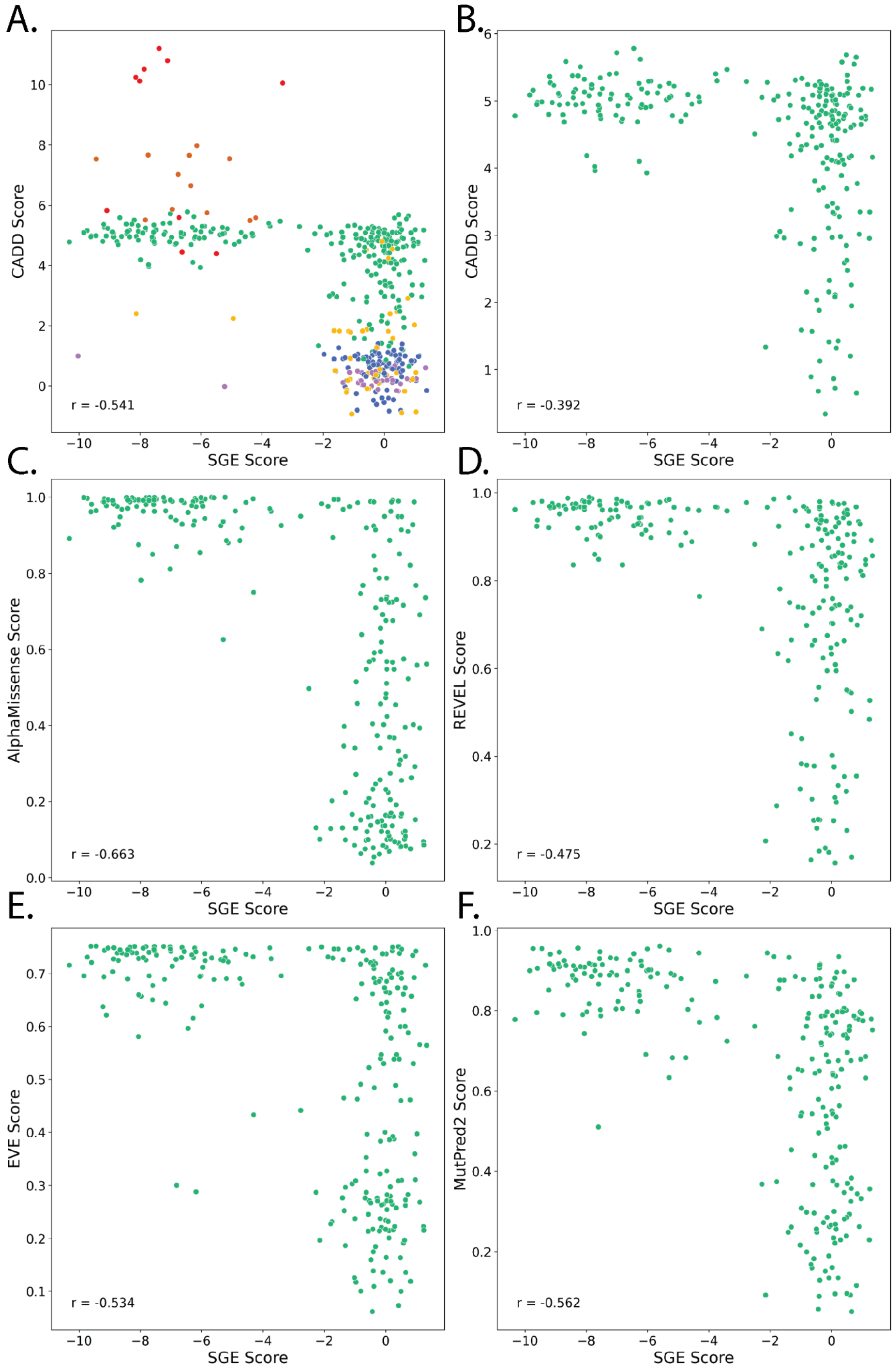

Supplement: 6 — Supplementary Figure 1: MYBPC3 iPSC-SGE repair templates. A) Reference background allele including meGFP fusion and puromyosin resistance cassette. Repair template has the 3’ homology arm deleted from the intronic sequence adjacent to exon 28. B) SNV allele repair template backbone that targets the deleted region from the background allele repair template that is still present in the wild type allele. The 3’ homology arm is also deleted from the intronic sequence adjacent to exon 28 to ensure HDR with the full repair template. SNVs were introduced into exon 32 or 33 and flanking intronic sequences. Supplementary Figure 2: POLG iPSC-SGE repair templates: A) Reference background allele including meGFP fusion and puromyosin resistance cassette. W748S repair template has the 3’ homology arm deleted from the intronic sequence adjacent to exon 11. Null repair template deletes exons 11-22 and fuses meGFP to in frame deletion product. B) SNV allele repair template backbone that targets the deleted region from the background allele repair template that is still present in the wild type allele. The 3’ homology arm is also deleted from the intronic sequence adjacent to exon 11 to ensure HDR with the full repair template. SNVs were introduced into exon 16 and flanking intronic sequences. Supplementary Figure 3: MYBPC3 iPSC-SGE cells. iPSC colony imaged after blasticidin selection for SNV allele integration. Cells were then differentiated to cardiomyocytes and dissociated and plated sparsely for imaging at differentiation day 20. Top row shows a representative wild type like cell with GFP and mApple signal localizing to sarcomeres. Bottom row shows a reduced abundance variant with normal GFP signal but very dim and diffuse mApple signal. Scale bar indicates 25 μm. Supplementary Figure 4: Disruption of MYBPC3 electrostatic potential causes reduced abundance. A) MYBPC3 C10 domain (gray backbone) with spheres representing charged residue beta carbons. The size of the sphere indicat [file NIHPP2025.11.12.25340127v1-supplement-6.pdf]
